# A Manual of Toxicology

**Published:** 1874-07

**Authors:** 


*A Manual of Toxicology, including the considertion of the Nature, Properties, Effects and Means of Detection of Poisons, more especially in their Medico-Legal Relations.* By John J. Reese, M. D. Philadelphia: J. B. Lippincott & Co., 1874, Buffalo: Martin Taylor.

The majority of medical men, when called upon to testify in court in a case of poisoning, are so profoundly ignorant of the subject, that it is painful to listen to their efforts to attempt to explain the lesions present, or the effect of the substance supposed to have been taken. And even when so called experts are placed upon the stand their testimony is so often given in a hesitating manner, and they so often contradict their own statements that the whole profession, is disgraced by the lack of intelligence.

This being the condition of affairs, the work of Dr. Reese should be received with gladness, for it presents in a compact and plain form the most essential elements of toxicology.

The first portion of the work gives, a general review of the subject of toxicology, considering what a Poison is, its mode of action, different modes of access to the organs of the body, causes of death by poisons, circumstances which modify poisons, Post-Mortem imbibition of poisons, Evidences of poisoning; by symptoms post mortem appearances, etc., Compound poisoning, Methods of chemical procedure, Medico-legal considerations, Duties and privileges of Medical experts, etc., etc. Part second gives a general classification of Poisons, for which purpose Dr. Reese adopts the physiological basis.

The author has carefully considered his subject, and no one can read his book without being fully impressed with the importance of toxicology. When it becomes generally known and read by the profession, we doubt very much if so many unqualified persons will be willing to go before the court and give evidence upon so important a subject as a case of poisoning.

The utter disagreement between the medical witnesses in several recent important trials has tended to throw odium upon the profession in general, and such will always be the case when unqualified persons are willing to go upon the stand and testify, in hopes notoriety or of a large fee

